# Low-level Circulation of Enterovirus D68–Associated Acute Respiratory Infections, Germany, 2014

**DOI:** 10.3201/eid2105.141900

**Published:** 2015-05

**Authors:** Janine Reiche, Sindy Böttcher, Sabine Diedrich, Udo Buchholz, Silke Buda, Walter Haas, Brunhilde Schweiger, Thorsten Wolff

**Affiliations:** Robert Koch Institute, Berlin, Germany

**Keywords:** enterovirus D68, EV-D68, enterovirus, viruses, respiratory infections, influenza-like illness, sentinel surveillance, Germany

## Abstract

We used physician sentinel surveillance to identify 25 (7.7%) mild to severe infections with enterovirus D68 (EV-D68) in children and adults among 325 outpatients with acute respiratory infections in Germany during August–October 2014. Results suggested low-level circulation of enterovirus D68 in Germany. Viruses were characterized by sequencing viral protein (VP) 1 and VP4/VP2 genomic regions.

Enterovirus D68 (EV-D68) belongs to the family *Picornaviridae*, genus *Enterovirus*, species *Enterovirus D*. Since its initial discovery in 1962 (*1*), EV-D68 infections in humans have been reported rarely. However, since 2008, clusters of acute respiratory infections (ARIs) associated with EV-D68 have been reported worldwide, including Europe (*2*–*5*).

During mid-August 2014–January 2015, the United States and Canada had nationwide outbreaks of EV-D68 infections associated with severe respiratory disease (*6*,*7*). The US Centers for Disease Control and Prevention and state public health laboratories confirmed 1,153 persons in 49 states and the District of Columbia infected by EV-D68 (*8*). Over the same period, >200 specimens tested were positive for EV-D68 throughout Canada (*7*). Clinical symptoms ranged from mild to severe disease requiring intensive care and mechanical ventilation. Children were predominantly affected, in particular if they had asthma or wheezing (*6*,*9*). After ARIs, symptoms of neurologic illness or acute flaccid myelitis developed in an increasing number of children (*10*,*11*).

To describe EV-D68 circulation in a large country in Europe in the fall of 2014, we investigated specimens from patients with respiratory illness for EV-D68. This investigation was conducted within the national outpatient ARI sentinel surveillance in Germany.

## The Study

Nasal swab specimens from outpatients with influenza-like illness (ILI), ARI, or both were collected by sentinel physicians participating in sentinel surveillance in Germany during weeks 32–44 in 2014 and sent to the Robert Koch Institute (Berlin, Germany). All specimens were tested in parallel for respiratory viruses, including influenza viruses A and B, rhinovirus/enterovirus, respiratory syncytial virus, adenovirus, and metapneumovirus by specific real-time reverse transcription PCRs (Technical Appendix). The rhinovirus/enterovirus real-time PCR detected rhinovirus at a limit of detection of 26 copies/reaction. EV-D68 was identified at a slightly lower sensitivity of 118 copies/reaction.

Rhinovirus/enterovirus–positive specimens were screened for EV-D68 by sequencing the viral protein (VP) 4/VP2 gene regions. Rhinovirus/enterovirus–negative specimens and samples without sequencing results were additionally analyzed by using a specific EV-D68 PCR (*12*). VP4/VP2 and VP1 regions were sequenced (GenBank accession nos. KP189380–KP189403 and KP657730–KP657747) for EV-D68–positive specimens.

**Figure 1:**
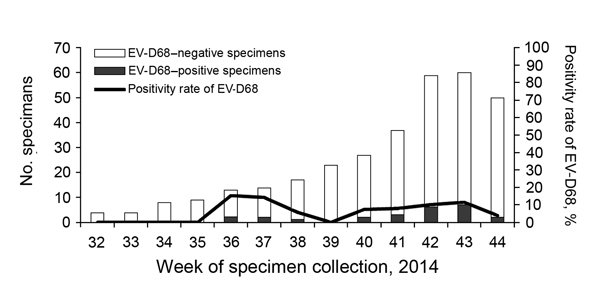
Detection of enterovirus D68 (EV-D68), Germany, week 32–44, 2014.

Rhinovirus/enterovirus was detected in 44% (143/325) of the specimens; 98 were identified as rhinovirus types A–C and 25 as EV-D68. The remaining 20 specimens could not be subtyped, but were negative for EV-D68 by using the specific PCR. The proportion of EV-D68 corresponded to 7.7% of the study collective. EV-D68 was initially detected from week 36 (August) through 38 (September) and continuously from week 41 through week 44 in October (Figure 1).

In addition to the other viruses tested, EV-D68–positive specimens were screened for parainfluenza virus 1–4, coronaviruses (NL63, OC43, HKU1, 229E), and bocavirus. None of these viruses was detected in EV-D68–positive patients, which suggested that EV-D68 played a major role in causing respiratory disease.

Major symptoms associated with EV-D68 infection included sudden onset, fever/shivers, cough, and sore throat (Table 1). Pneumonia was diagnosed in a 7-year-old boy and a 10-year-old girl, and a 2-year-old girl was hospitalized because of obstructive bronchitis. For 11 (44%) of 25 case-patients, a concurrent chronic condition was reported: 5 with a respiratory condition, 3 with a cardiac condition, 2 with diabetes, and 1 with a neurologic disorder. EV-D68 was detected in 10 children and 15 adults; 56% of these patients were male.

**Table 1:** Demographic and clinical characteristics of 25 patients infected with enterovirus D68, Germany, weeks 32–44, 2014*

Patient	Week†	Age, y/sex	Federal state	Sudden onset of disease	Fever/shivers	Cough	Sore throat	Clinical follow-up	Admission to hospital	Underlying condition
1	36	6/F	Thuringia	+	+	+	+	ILI	No	None
2	36	42/M	North Rhine-Westphalia	+	+	+	+	ILI	No	Respiratory system
3	37	61/M	North Rhine-Westphalia	+	+	+	+	ILI	No	Cardiac system
4	37	42/F	North Rhine-Westphalia	–	+	+	+	ARI	No	Respiratory system
5	38	11/M	Thuringia	+	+	+	+	ILI	No	None
6	40	35/F	Lower Saxony	+	+	+	–	ILI	No	Respiratory system, diabetes
7	40	53/M	North Rhine-Westphalia	+	+	+	+	ILI	No	Cardiac system
8	41	2/M	Thuringia	+	+	+	–	ILI	No	None
9	41	62/M	Hesse	+	–	+	+	ARI	No	None
10	41	7/M	Rheinland-Palatinate	+	+	+	+	Pneumonia	No	Respiratory system
11	42	8/F	Bavaria	NA	NA	+	+	ARI	No	None
12	42	25/M	Hesse	+	–	+	–	ARI	No	None
13	42	22/M	Lower Saxony	+	+	+	+	ILI	No	None
14	42	14/F	Baden-Württemberg	–	–	+	+	ARI	No	None
15	42	43/M	Bavaria	+	–	+	+	ARI	No	None
16	42	10/F	Berlin	–	+	+	+	Broncheal pneumonia	No	Respiratory system
17	43	3/F	North Rhine-Westphalia	+	+	+	–	ILI	No	None
18	43	12/F	Bavaria	+	–	+	+	ARI	No	None
19	43	52/M	Lower Saxony	+	+	+	+	ILI	No	Diabetes
20	43	26/M	North Rhine-Westphalia	+	+	+	–	ILI	No	None
21	43	44/M	North Rhine-Westphalia	+	–	+	+	ARI	No	Cardiac system
22	43	2/F	Hesse	+	+	+	–	Obstructive bronchitis	Yes	None
23	43	41/M	Saarland	+	+	+	+	ILI	No	None
24	44	2/F	Schleswig-Holstein	+	–	+	–	ARI	No	None
25	44	73/F	North Rhine-Westphalia	–	–	+	+	ARI	No	Neurologic disorder

*+, positive; ILI, influenza-like illness; –, negative; ARI, acute respiratory infection; NA, not available. †Data are listed by week of symptom onset.

Patients infected with EV-D68 came from different federal states in Germany; no epidemic cluster or outbreak was detected in the context of these patients. Syndromic surveillance data of corresponding sentinel practices showed only a partial coincidence of EV-D68–positive patients and an increase of ARI activity in the practice. However, the increase in ARI activity was probably caused by unrelated RV activity.

Sequence analysis is not regularly performed for rhinovirus/enterovirus–positive specimens within sentinel surveillance in Germany. However, comparative data can be provided for week 36 through week 20 for the 2009–10 and 2010–11 seasons (Table 2). During those seasons, patients with ILI in 5 age groups (<1–4, 5–15, 16–34, 35–60, and >60 years) were investigated by using the rhinovirus/enterovirus real-time reverse transcription PCR. Within the seasons, an average of 21% (198/952 for 2009–10) and 13% (128/1002 for 2010–11) of specimens were positive for rhinovirus/enterovirus (Table 2). At least 20% of the rhinovirus/enterovirus–positive specimens were arbitrarily chosen for sequencing (mainly RV A, B, or C; 1 echovirus), but no EV-D68 was identified.

**Table 2:** Detection of rhinovirus and enterovirus by national outpatient ARI sentinel surveillance, Germany, weeks 36–20, 2009–2010 and 2010–2011*

Age group, y	No. specimens tested	No. rhinovirus/enterovirus–positive specimens (%)	No. rhinoviruses/enteroviruses sequenced	No. rhinoviruses detected	No. enteroviruses detected
2009–2010					
<1–4	156	45 (29)	11	11	0
5–15	386	71 (18)	18	17	1†
16–34	225	40 (18)	12	12	0
35–60	157	34 (22)	19	19	0
>60	28	8 (29)	4	4	0
Total	952	198 (21)	64	63	1
2010–2011					
<1–4	271	56 (21)	21	21	0
5–15	363	40 (11)	20	20	0
16–34	189	20 (11)	17	17	0
35–60	153	12 (9)	11	11	0
>60	26	0 (0)	0	0	0
Total	1,002	128 (13)	69	69	0

*ARI acute respiratory infection.  †Echovirus 9.

**Figure 2:**
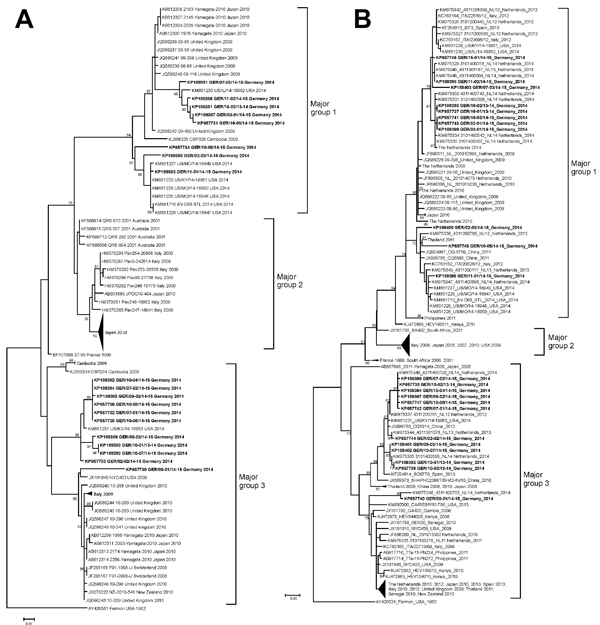
Phylogenetic analysis of selected enterovirus D68 (EV-D68) strains based on nucleotide sequences of A) partial viral protein (VP) 4/VP2 region (357 nt) corresponding to nt 733–1089 in the Fermon strain (GenBank accession no. AY426531); and B) partial VP1 region (339 nt) corresponding to nt 2521–2859 in the Fermon strain. Trees were constructed by using maximum-likelihood estimation (Tamura 3-parameter with 5 gamma distributed rates among sites) with 1,000 replicates through MEGA 5.2 (http://www.megasoftware.net/). The Fermon strain was used as an outgroup. Reference sequences were selected from the United States, countries in Europe, and other regions, mainly during 2005–2014. Selected reference sequences are not identical in both trees because complementary VP1 and VP4/VP2 sequence data are not available for all reference viruses. Major groups 1, 2, and 3 are shown as described by Meijer et al. (*13*,*14*). Only bootstrap values >65% are shown at branch nodes. EV-D68 strains from Germany are indicated in bold. Scale bar indicate nucleotide substitutions per site. Some parts of the trees are collapsed. For an expanded version of Figure 2, sees the Technical Appendix.

Phylogenetic analysis of EV-D68 strains detected in Germany was conducted for the VP1 and the VP4/VP2 genomic regions (Figure 2). Analysis placed EV-D68 isolates from Germany into major groups 1 or 3 and clustered with strains from the United States and the Netherlands from 2014, which indicated circulation of similar strains in the United States and Europe.

## Conclusions

In the 2014 outbreak in the United States, ≈36% (2,600) of specimens were positive for EV-D68; children were predominantly affected. Because testing was prioritized for children with severe respiratory illness, there were probably more cases of mild infections (*8*). Information on EV-D68 circulation during this period for Europe is rare. This finding might be caused by insufficient sampling of patients with ARI or limited detection of EV-D68 by laboratory diagnostics (*9*). Sporadic cases of neurologic disease after EV-D68 infection were reported from France and the United Kingdom (*9*,*10*).

Investigation for EV-D68 has been continuously performed in the Netherlands since the increase in infections in 2010 (*13*). The ILI/ARI sentinel system in the Netherlands identified 24 EV-D68 infections in 2010, none in 2011, 7 in 2012, 3 in 2013, and 5 in 2014 (by week 40) (*13*,*14*), which probably increased toward the end of that year (*9*). For the 2014 season, a hospital in the Netherlands reported an increase of EV-D68; 16 patients were infected (*12*). Such an increase in EV-D68 infections was already seen in 2010 at the same hospital along with an increased number of cases throughout the country (*13*). This finding increased the possibility that an increase in EV-D68 infections in primary care will also extend to increased numbers of infections in patients in secondary care. So far, we report low EV-D68 circulation in Germany: 25 sporadic cases in 2014.

Clinical patterns in patients in Germany were largely determined by the ILI/ARI case definition for collecting specimens. Most (88%) patients had mild disease. Severe disease was observed in 3 children who had obstructive bronchitis and pneumonia, and 1 child required hospital care. Similarly, mild respiratory disease was predominantly observed for patients in the Netherlands (*14*). However, more severe cases were detected among hospitalized children who had life-threatening respiratory illness, as described in the United States (*6*,*12*,*14*). More than half of patients with severe respiratory illness in Germany and the Netherlands had concurrent conditions (*12*,*14*), which seem to be a major factor for development of severe disease after EV-D68 infection (*6*).

Phylogenetic analysis of EV-D68 from Germany showed high similarity with current strains from the United States and the Netherlands (*12*,*14*). These new genetic clusters reflect the evolution of EV-D68 and might be associated with an increasing activity of this virus. For an improved understanding of the factors determining local and temporal differences in respiratory disease outbreaks, continuous collection of global data by representative surveillance systems is needed.

**Technical Appendix.** Supplemental methods used for detection of enterovirus D68–associated acute respiratory infections, Germany, 2014.
