# Supplementary material for: Low-level Circulation of Enterovirus D68–Associated Acute Respiratory Infections, Germany, 2014
Source: Emerg Infect Dis. 2015 May;21(5):837–41. doi: 10.3201/eid2105.141900 (PMC4412236; doi:10.3201/eid2105.141900)
Supplement: Supplementary file 1 — Technical Appendix. Supplemental methods used for detection of enterovirus D68–associated acute respiratory infections, Germany, 2014. [file 14-1900-Techapp-s1.pdf]

Article DOI: http://dx.doi.org/10.3201/eid2105.141900

Low-level Circulation of Enterovirus D68–Associated Acute Respiratory Infections, Germany, 2014

Technical Appendix

Clinical Specimens and Ethical Statement

Specimens were collected from patients with influenza-like illness/acute respiratory infection by physicians participating in national outpatient acute respiratory infection sentinel surveillance in Germany (*1*). Sampling was performed only for patients who provided written consent for laboratory examination and for collection of patient-specific data (date of disease onset, sampling date, symptoms, and underlying chronic illness). Specimens were sent to the National Reference Center for Influenza (Berlin, Germany) for national surveillance of influenza and acute respiratory infections in Germany. Analyses of all data were conducted anonymously. National virologic surveillance of influenza and other respiratory viruses was approved by the Ethics Committee of the Charité, Humboldt University, Berlin (EA2/126/11).

Real-Time PCR for Rhinovirus/Enterovirus (EV) and Specific EV-D68 Detection

RNA was extracted from 300-μL specimens by using the MagAttract Viral RNA M48 Kit (QIAGEN, Hilden, Germany) and eluted in 80 μL of elution buffer. Alternatively, RNA was extracted by using the RTP DNA/RNA Virus Mini Kit (Invitek, Berlin, Germany) with 400-μL specimens and an elution volume of 60 μL. Twenty-five microliters of extracted RNA were subjected to cDNA synthesis by using 200 U M-MLV reverse transcriptase (Invitrogen, Karlsruhe, Germany) in a total volume of 40 μL (*2*). The rhinovirus/enterovirus real-time PCR uses primer complementary to regions within the 5′-untranslated region (corresponding to nt 351–558 in the Fermon strain; GenBank accession no. AY426531) and detects rhinovirus with a limit of detection of 26 copies/reaction. This method also detects enterovirus, including strains CV-A9, CV-A10, CV-A16, CV-A21, CV-A24, CV-B1–6, E-4, E-9, E-11, E-20, E-21, E-25, EV-B69, and EV-D68. The limit of detection for EV-D68 was 118 copies/reaction.

Rhinovirus/enterovirus PCR was performed in a 25-μL reaction mixture that contained 3 μL of cDNA product, 300 nmol/L of primer pairs rhinovirus/enterovirus–375-F1 (5′-GTG

KYC YAG CCT GCG TGG C-3′) and rhinovirus/enterovirus–586-R1 (5′-ACG GAC ACC CAA AGT AGT YGG T-3′), 100 nmol/L of probe rhinovirus/enterovirus–476 (5′-YAK-CCT CCG GCC CCT GAA TGY GGC TAA–BBQ-3′), 0.1 mmol/L dNTP (Invitrogen) with dUTP (GE Healthcare, Munich, Germany), 5 mmol/L $MgCl_2$ (Invitrogen), 0.5 U Platinum *Taq* DNA polymerase (Invitrogen), and PCR buffer (200 mmol/L Tris-HCl, pH 8.4, 500 mmol/L KCl). Amplification was conducted at 95°C for 5 min, followed by 45 cycles at 95°C for 15 s and 60°C for 30 s. All reactions were conducted by using an ABI PRISM 7500 Sequence Detection System (Applied Biosystems, Weiterstadt, Germany) or LightCycler 480 Real-Time PCR System (Roche Deutschland Holding GmbH, Gernzach, Germany), respectively.

The protocol for detection of EV-D68 was adapted from that of Poelman et al. (*3*). This protocol uses 300 nmol/L of primer pairs EV-D68 FW (5′-TGT TCC CAC GGT TGA AAA CAA-3′) and EV-D68 RV (5′-TGT CTA GCG TCT CAT GGT TTT CAC-3′), and 150 nmol/L of probe EV-D68 (5′-6FAM-WCC GCT ATA GTA CTT CG-MGB-3′) in a volume of 25 μL in a 2 step real-time PCR.

Real-Time PCR for Other Respiratory Pathogens

In addition to detection of rhinovirus/enterovirus, all specimens were tested for influenza A and B viruses, respiratory syncytial virus, human metapneumovirus, and adenovirus by using in-house real-time PCRs as described (*2*,*4*–*6*). EV-D68–positive specimens were further screened for parainfluenza virus 1–4, coronaviruses (NL63, OC43, HKU1, and 229E), and bocavirus by using 2-step real-time PCRs (oligonucleotide sequences can be provided on request).

Amplification of Viral Protein (VP) 4/VP2 Genome Regions

From rhinovirus/enterovirus–positive specimens, amplification of the VP4/VP2 was performed with 4 μL of cDNA in a 50-μL reaction by using 600 nmol/L of primer rhinovirus/enterovirus–Seq-F2-Out (5′-CGG CCC CTG AAT GCG GCT AA-3′), 900 nmol/L of primer rhinovirus/enterovirus–Seq-9565-R (5′-GCA TCI GGY ARY TTC CAC CAN CC-3′) (*7*), 0.1 mmol/L dNTP (Invitrogen) with dUTP (GE Healthcare), 2 mmol/L mM $MgCl_2$, 0.5 U Platinum *Taq* DNA polymerase (Invitrogen), and PCR buffer (200 mmol/L Tris-HCl, pH 8.4, 500 mmol/L KCl). Amplification was conducted at 94°C for 5 min; followed by 40 cycles at 94°C for 30 s, 65°C for 30 s, and 72°C for 1 min; and a final extension at 72°C for 10 min. Amplified products (634 bp) were analyzed by electrophoresis on a 1.5% agarose gel.

For samples that had negative results, 4 μL of the external PCR sample was used for a seminested PCR, which was performed in a 50-μL reaction with 300 nmol/L of rhinovirus/enterovirus–Seq-9895-F (5′-GGG ACC AAC TAC TTT GGG TGT CCG TGT-3′) (*7*) and rhinovirus/enterovirus–Seq-9565-R, respectively. The cycling protocol was the same as that for the external PCR except that the annealing temperature was 60°C. Nested amplicons (549 bp) were visualized by using agarose gel electrophoresis.

Sequencing of the VP4/VP2 region was performed for at least 20% of the rhinovirus/enterovirus–positive specimens of all age groups in the seasons 2009/2010 and 2010/2011, respectively. For the 2014 season, sequencing was performed from all specimens, identifying RV A-C and EV-D68. All EV-D68-positive samples were further subjected to amplification of the VP1 gene (online Technical Appendix Table).

Amplification of VP1 Genome Region

VP1 was amplified by using the One-Step-Reverse Transcription PCR Kit (QIAGEN), followed by a nested PCR with HotStarTaq-Mastermix (QIAGEN). Reverse transcription PCR was conducted in a 12.5-μL reaction that contained 2 μL of RNA, 600 nmol/L of primer NRZ 267 (5′-ATG YTA GST ACW CAT RTB GTB TGG GAY TT-3′), 600 nmol/L of NRZ 268 (5′-ATC CAY TGR ATM CCW GGG CCY TCR AAR C-3′) according to the manufacturer’s protocol. The temperature profile used was 22°C for 10 min, 50°C for 45 min, and 95°C for 15 min, followed by 40 cycles at 94°C for 30 s, 55°C for 30 s, and 72°C for 90 s. Final elongation conducted at 72°C for 10 min.

Nested PCR was conducted by using 1 μL of reverse transcription PCR samples in a 12.5-μL volume that contained 600 μmol/L of primer NRZ 269 (5′-AAT GCY AAY GTT GGY TAY GTY ACH TGT T-3′) and 600 μmol/L of primer NRZ 270 (5′-AAG AYC CYA CAA ARA CYC CHC CRW ARC CKG G-3′). Amplification was conducted by using a touchdown protocol with 10 cycles (94°C for 30 s, 60°C for 30s, and 72°C for 90 s) 10x with a decrease of 1°C/cycle in the annealing temperature, followed by 30 cycles (94°C for 30 s, 50°C for 30 s, and 72°C for 90 s), and final elongation 72°C for 10 min. The resulting amplification product (1,129 bp) was visualized by electrophoresis on a 1.5% agarose gel.

Sequencing

VP4/VP2 amplification products were purified either directly by using the MSB SpinPCRapace Kit (Stratec Molecular, Birkenfeld, Germany) or from agarose gels by using the Invisorb Spin DNA Gel Extraction Kit (Thermo Scientific, Schwerte, Germany) according to manufacturer’s instructions. Purified PCR products were cycle sequenced in the forward

and the reverse directions with primer pairs rhinovirus/enterovirus–Seq-F2-Out and rhinovirus/enterovirus–Seq-9565-R or rhinovirus/enterovirus–Seq-9895-F and rhinovirus/enterovirus–Seq-9565-R, respectively, in a 3130xl Genetic Analyzer (Applied Biosystems, Foster City, CA, USA) by using the BigDye Terminator v3.1 Cycle Sequencing Kit (Applied Biosystems).

VP1 amplification products were directly purified by using ExoSAP-IT (Affymetrix, High Wycombe, UK). Sequence reaction was conducted by using primers NRZ 269, NRZ 270, and NRZ 271 (5′-CAA GCA ATG TTY GTA CCH ACT GG-3′).

References

1. der Heiden MA, Kopke K, Buda S, Buchholz U, Haas W. Estimates of excess medically attended acute respiratory infections in periods of seasonal and pandemic influenza in Germany from 2001/02 to 2010/11. PLoS ONE. 2013;8:e64593. PubMed http://dx.doi.org/10.1371/journal.pone.0064593

2. Reiche J, Schweiger B. Genetic variability of group A human respiratory syncytial virus strains circulating in Germany from 1998 to 2007. J Clin Microbiol. 2009;47:1800–10. PubMed http://dx.doi.org/10.1128/JCM.02286-08

3. Poelman R, Scholvinck EH, Borger R, Niesters HG, van Leer-Buter C. The emergence of enterovirus D68 in a Dutch university medical center and the necessity for routinely screening for respiratory viruses. J Clin Virol. 2015;62:1–5. PubMed http://dx.doi.org/10.1016/j.jcv.2014.11.011

4. Schulze M, Nitsche A, Schweiger B, Biere B. Diagnostic approach for the differentiation of the pandemic influenza A(H1N1)v virus from recent human influenza viruses by real-time PCR. PLoS ONE. 2010;5:e9966. PubMed http://dx.doi.org/10.1371/journal.pone.0009966

5. Chmielewicz B, Nitsche A, Schweiger B, Ellerbrok H. Development of a PCR-based assay for detection, quantification, and genotyping of human adenoviruses. Clin Chem. 2005;51:1365–73. PubMed http://dx.doi.org/10.1373/clinchem.2004.045088

6. Reiche J, Jacobsen S, Neubauer K, Hafemann S, Nitsche A, Milde J, et al. Human metapneumovirus: insights from a ten-year molecular and epidemiological analysis in Germany. PLoS ONE. 2014;9:e88342. PubMed http://dx.doi.org/10.1371/journal.pone.0088342

7. Savolainen C, Blomqvist S, Mulders MN, Hovi T. Genetic clustering of all 102 human rhinovirus prototype strains: serotype 87 is close to human enterovirus 70. J Gen Virol. 2002;83:333–40. PubMed

Technical Appendix Table. Overview and accession numbers of enterovirus D68 sequences by genome region, Germany, 2014*

Specimen†	Accession no., VP4/VP2	Accession no., VP1
GER/16-01/13-14	–	KP657737
GER/10-01/13-14	KP189380	KP189392
GER/10-02/13-14	–	KP657738
GER/10-03/13-14	–	KP657739
GER/16-02/13-14	KP189381	KP189393
GER/09-01/14-15	KP657730	KP657740
GER/10-04/14-15	KP189382	KP189394
GER/16-03/14-15	KP657731	KP657741
GER/07-01/14-15	KP657732	KP657742
GER/11-01/14-15	KP189383	KP189395
GER/02-01/14-15	–	KP657743
GER/07-02/14-15	KP189384	KP189396
GER/09-02/14-15	KP189385	KP189397
GER/11-02/14-15	KP189386	KP189398
GER/02-02/14-15	KP657733	KP657744
GER/03-01/14-15	KP189387	KP189399
GER/10-05/14-15	KP657734	KP657745
GER/02-03/14-15	KP189388	KP189400
GER/09-03/14-15	KP189389	KP189401
GER/10-06/14-15	KP657735	–
GER/10-07/14-15	KP189390	KP189402
GER/15-01/14-15	–	KP657746
GER/07-03/14-15	KP189391	KP189403
GER/12-01/14-15	–	–
GER/10-08/14-15	KP657736	KP657747

*VP, viral protein; –, no sequence was obtained.
†Sequencing of the VP4/VP2 and the VP1 regions was performed with enterovirus D68–positive specimens. Of the 25 specimens, sequences were obtained from 24 specimens, mainly for both genome regions. For 1 specimen (GER/12-01/14-15), no sequencing result was obtained.

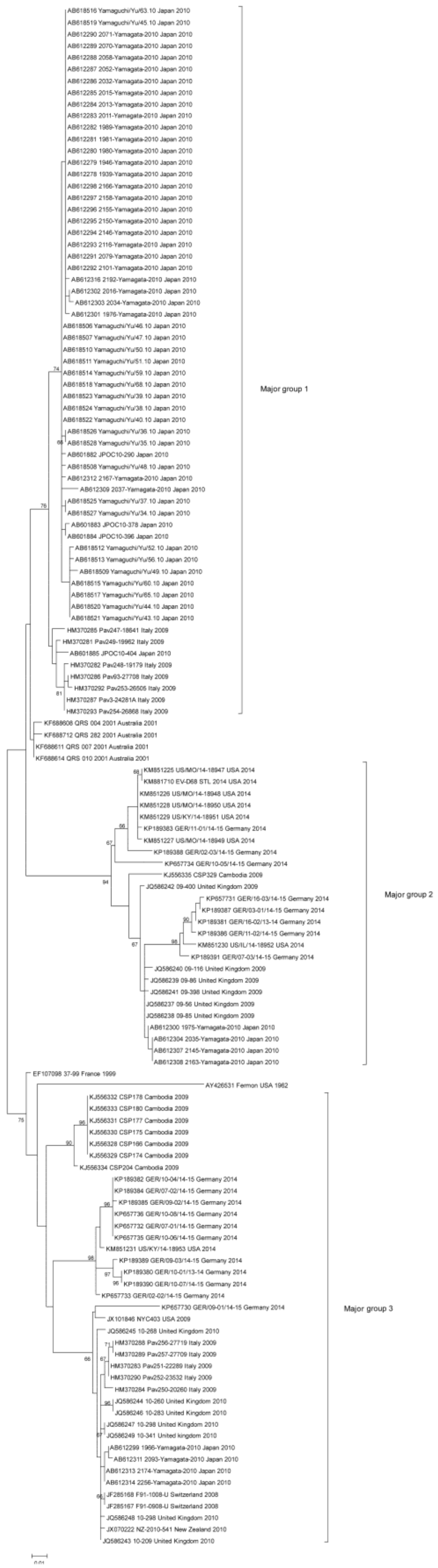

Technical Appendix Figure 1. Expansion of phylogenetic analysis of viral protein (VP) 4/VP2 (uncollapsed) of enterovirus D68, Germany, 2014. Scale bar indicates nucleotide substitutions per site.

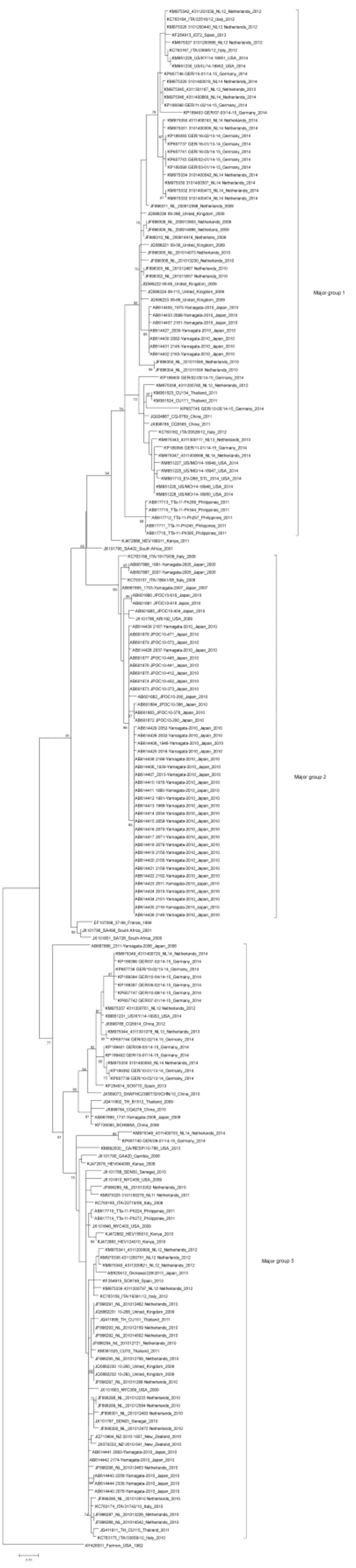

Technical Appendix Figure 2. Expansion of phylogenetic analysis of viral protein (VP) 1 (uncollapsed) of enterovirus D68, Germany, 2014. Scale bar indicates nucleotide substitutions per site.
